# Trade vulnerability assessment in the grain-importing countries: A case study of China

**DOI:** 10.1371/journal.pone.0257987

**Published:** 2021-10-22

**Authors:** Jian Duan, Yong X. U., Haining Jiang

**Affiliations:** 1 College of Geography and Environmental Sciences, Zhejiang Normal University, Jinhua, China; 2 Key Laboratory of Regional Sustainable Development Modeling, CAS, Beijing, China; 3 Institute of Geographic Science and Natural Resources Research, CAS, Beijing, China; Szechenyi Istvan University: Szechenyi Istvan Egyetem, HUNGARY

## Abstract

Since the 2008 global food crisis, food security vulnerability has been a prominent topic in the food policy debate. However, vulnerability is inherently difficult to conceptualize and is more challenging to operationalize and measure. This study constructs a mathematical model and takes China as a case study to measure the vulnerability and sensitivity of China with its partners in the international grain trade. The results show that 1) the degree of interdependence between China and its grain trading partners is asymmetric, which generates trade vulnerability or economic power; 2) the vulnerability of China’s food trade shows a high spatiotemporal heterogeneity among countries: the higher vulnerability zones are concentrated in North America and Northeast Asia, and the scope of the higher vulnerability zones tends to expand; 3) Our results also reveal that China also has different sensitivities to fluctuations in grain markets from different countries, and the higher sensitive zones of the grain trade in China are mainly distributed in America, Europe, and Oceania. The main contribution of this paper is the development of a methodology for food trade vulnerability assessment and examines the influence of international food trade on food security in importing countries, measured using the vulnerability index and sensitivity index. Nevertheless, the conclusions of this study can be considered preliminary, and there remains great potential for future studies to deepen and broaden our analyses further.

## Introduction

Food security has been a long-standing concern worldwide [1]. There were still 690 million people suffering from hunger in the world in 2019, an increase of nearly 60 million compared to 2015 [2]. Feeding the world’s population is a challenge that is likely to increase in the future. The global population is expected to reach 9 billion by 2050, adding two billion mouths to the current population [3, 4]. In 2015, the FAO set a goal of achieving zero hunger by 2030.Hunger is caused by inadequate food supplies, and worldwide trade underpins food security by distributing food surpluses to food-deficient countries [5]. Food trade provides an alternative means of achieving this goal [1, 6–8]. It also provides a buffer against local variability in food resources because regions can import when they have a deficit and export when they have a surplus [5]. The trade of agricultural products has increased dramatically over the past decades [1, 4, 5]. For example, the amount of cereal exports has increased from 79 to 480 million tons since 1961 [9]; the rapidly increasing international food trade has drastically altered the global food system over the past decades. Today, approximately 23% of total agricultural production is subject to international trade, about one-quarter of the food produced for human consumption is traded internationally, and about 5.1 billion people are estimated to live in a net food importing country [5, 10].

However, the expansion of global food markets brings benefits but also risks, such as increased reliance on trade, which increases vulnerability to food shortages in the real-world trading network [1, 5, 6, 10]. Food supply diversity has increased significantly in most of the world’s population at the cost of a high dependency on food imports. Despite a growing number of people heavily dependent on imports, the number of import partners has decreased more often than it has increased [8]. The world’s main grain export markets are concentrated in North America, the Black Sea region, and South America. As of 2014, 2/3 of global agricultural exports came from 12 countries or regions; each species was dominated by only a few countries [11]. This combination of increased dependency on imports and a reduced number of import partners indicates a potential vulnerability to disruptions in linked food systems [8]. While food supply diversity increased for most of the world, it came with the cost of increased trade dependency, potentially exposing many of these countries to shocks in the few major exporting countries [8]. Particularly for developing countries, many developing countries rely on imports for food consumption. They are always at risk of high vulnerability to rising prices of agricultural products and are highly exposed to demand shocks coming from their less diversified export markets and internal shocks based on dependence upon strategic imports, not only from an economic point of view but also to political and other exogenous shocks [8, 12].

For instance, this interdependence of regions for food resources has been observed during the food price crises of 2007–2008 and 2010–2011. Price surges were induced by a combination of extreme weather and environmental events (e.g., wildfires and droughts), which led to a reduction in food prices in global markets, leading to an increase in food prices for trading nations [6]. Thus, since the 2008 global food crisis, food security vulnerability has been a prominent topic in the food policy debate [2]. Recently, COVID-19 has severely disrupted the global food production and trading system, making the global food supply system more vulnerable, and governments are actively acting to increase their food systems [13, 14]. As a widely used analytical tool, "vulnerability" can provide predictive and regulatory measures for food security assessment and governance.

## Literature review

### Vulnerability research

The concept of vulnerability was used in the 1970s by geographers and social scientists in risk management to describe the fragility of certain communities or countries facing severe environmental or socio-economic risks, such as earthquakes or food exchange crises [15]. In the decade after 2000, the use of the vulnerability concept increased sharply with its adoption by the Intergovernmental Panel on Climate Change to assess the potential impacts of global warming at regional and global levels [15]. Vulnerability later became a central focus of the global change science research community to discuss and define adaptation and mitigation plans. Scholars have developed a variety of methods to study and quantify the vulnerability of systems, including the risk-hazard model, pressure and release model, and vulnerability assessment framework [16]. This framework usually distinguishes between three distinct elements: level of exposure, level of sensitivity, and adaptive capacity [15].

Vulnerability has recently been widely applied in many research fields, such as disaster management, ecology, public health, climate change, land use, sustainability science, economics, and engineering. There is an increasing number of studies on climate change vulnerability [17–20], road network vulnerability [21], economic system vulnerability [22–25], social system vulnerability [26], urban vulnerability [27–29], and food security vulnerability [30–34]. Due to different research objects and disciplinary perspectives in different application fields, scholars hold great differences in the concepts of "vulnerability" vulnerability and vulnerability assessment [35, 36]. For instance, the literature on interdependence considers vulnerability in terms of the relative ease with which a state can adjust to the loss of a given trade tie [29]. Based on the economic interdependence theory, Du et al. constructed an evaluation model of the asymmetry of the sensitivity and vulnerability of trade and investment between interaction countries [25].

### Food security vulnerability assessment

Various studies have explored vulnerability in agricultural and food systems, divided into macro and micro perspectives. From a macro perspective, the vulnerability of agricultural systems has mainly been studied with regard to exposure to climatic perturbations, such as temperature changes, drought, or floods [20]. It has also been used to describe agricultural systems’ response to diverse socio-economic changes, such as market fluctuations or land use changes [30]. Dupraz1andBourdon used the Herfindahl-Hirschman index(HHI) and the Bonilla index(BI) to evaluate the vulnerability of food security to trade in 39 developing countries, demonstrating that trade concentration increases a country’s sensitivity to external shocks, which can have a negative or positive impact on relevant sectors and the foreign trade effects of the economy [12]. A study on the vulnerability of European Union Economies in Agro trade confirmed the EU heterogeneity of the vulnerability to global shocks in the agricultural sector. Certain general features suggest this differentiation, including the size of the economy, geographical location, maturity of the economy, and productivity of the agricultural sector [37]. From a macro perspective, scholars have focused on assessing household food security. Iboketal developed a vulnerability to food insecurity index to measure household vulnerability to food insecurity [34], and found that households with diversified livelihoods had higher resilience and adaptability than households with single livelihoods did.

However, few studies have explored the trade vulnerability of food systems with a political-economic dimension. This study examines the vulnerability of the food trade embedded in dependency theories. It is also expected to develop indicators and methods for the quantitative measurement of trade vulnerability. It becomes more important to understand the role of vulnerability in the onset and outcome of economic coercion. However, vulnerability is inherently difficult to conceptualize and is more challenging to operationalize and measure [24, 38]. This study constructs a mathematical model and uses China as a case study to measure the vulnerability and sensitivity of China with its partners in international grain trade; China is the largest food consumer and food importer country in the world, and its scale of grain imports increased by 108 million in 2018 from more than 43 partners.

The remainder of this paper is organized as follows. Section 3 introduces the research design and data sources. Section 4 focuses on the empirical analysis and provides a reasonable explanation of the results. Conclusions and implications for further research are discussed in Section 5.

## Research methods

This assumption follows naturally from the literature on interdependence, which centers on varying levels of economic dependence and their impact on states’ status and mobility [39, 40]. The concept of interdependence describes the process of interconnection, mutual influence, and interaction between state actors in political and economic aspects. With the development of global trade and investment, interdependence among nations has become a fundamental feature of the contemporary international community. National survival and well-being depend on the interaction’s power and influence [41].

The literature on interdependence considers vulnerability in terms of the relative ease with which a state can adjust to the loss of a given trade tie [39, 42]. Research has shown that a state’s position in the global trade network influences its vulnerability to its trade partners [24]. When a state’s value to trade partners is low, but its trade partners are very well connected to the broader global trade network, it is more likely to face asymmetrically high opportunity costs if that trade is terminated because its trade partners can more easily replace lost trade [20]. On the contrary, a state with little value to trade partners highly connected to the broader global trade network is more likely to acquire sanction threats. As such, when political disputes arise, leveraged states can inflict economic costs on targets while enduring relatively light costs themselves [24].

Two common usages of interdependence pervade the literature: “sensitivity interdependence” reflects the mutual effects of a relationship, and “vulnerability interdependence” is the opportunity cost of disrupting it [42]. Thus, vulnerability and sensitivity are two basic variables that characterize the interdependence relationship in research [43]. In our research, vulnerability refers to the extent to which disadvantaged actors suffer losses due to the costs imposed by external events, that is, the cost of their adjustments in response to external changes. Sensitivity refers to the degree of response within a certain policy framework, that is, how fast a country’s policy changes lead to costly changes in another country and what price is paid. Our hypotheses are:

Hypothesis 1: In the field of trade, a party with low dependence has power and can implement influence and control, while a party with a high degree of dependence is more vulnerable.
Hypothesis 2: Asymmetric dependence between countries is mainly determined by the market’s relative share and substitutability. A party with a higher market share is less dependent and difficult to replace. The party with a lower relative market share is more dependent on the other party, and another country can easily replace it.
Hypothesis 3: In the field of trade, certain trade interruptions or commerce restrictions have asymmetric effects. The extent of the impact is related to the total trade value of the countries. The party with a higher total trade value faces fewer effects, and the party with a lower total trade value suffers more.

### Mathematic models

Trade vulnerability is characterized by comparing the asymmetric dependence in bilateral trade. The vulnerability index(*V_ij_*) is constructed in two steps: first, indicators of import dependence(*I*_*ij*_) and export dependence(*E*_*ij*_) are used to characterize the asymmetric dependence in bilateral food trade between countries *i* and *j*; then, divide import dependence(*I*_*ij*_) by export dependence(*E*_*ij*_) to calculate the vulnerability index (*V_ij_*). The calculation formula is as follows:

$$I_{ij}=W_{ij}/\sum_{i=1}^{n}W_{ij}$$ (1)

$$E_{ij}=U_{ij}/\sum_{j=1}^{m}U_{ij}$$ (2)

$$V_{ij}=I_{ij}/E_{ij}$$ (3)

Where *V*_*ij*_ is the vulnerability index between countries *I* and *j*, *I*_*ij*_ denotes *i*’s import dependency on country *j*, *W*_*ij*_ is the amount of food *i* imports from country *j*, *E*_*ij*_ indicates country *i*’s export dependency on *j*. *U*_*ij*_ is the amount of grain exports from country *i* to*j*; *n* refers to the total number of countries or regions from which *i*’s imports come from,*and m* refers to the total number of countries where country *i*’s grain exports.

The values of *I*_*ij*_ and *E*_*ii*_ range in [0, 100%], and the greater the value, the higher the degree of independence. If *V*_*ij*_> 1, it indicates that *i* has less trade complementarity than *j*in grain trade, and the greater the value, the higher the degree of vulnerability. If *V*_*ij*_< 1, the vulnerability is low, and the smaller the value, the lower is the degree of vulnerability.

By comparing the asymmetric effects of changes in bilateral trade flows on both sides of the trade, trade sensitivity is characterized, and a sensitivity index is constructed. The calculation formula is as follows:

$$S_{ij}=\frac{\Delta T_{ij}}{\Delta T_{i}}/\frac{\Delta T_{ij}}{\Delta T_{j}}$$ (4)

In this expression, *S*_*ij*_ is the sensitivity index of country *i* to country *j*, *ΔT*_*ij*_ denotes the increase in the bilateral grain trade volume between *i* and *j* during the inspection period, *and ΔT*_*i*_ and *ΔT*_*j*_ represent the total grain imports and increase in total grain exports of country *i* and country *j* during the inspection period. The larger the absolute value of *S*_*ij*_, the higher the degree of sensitivity of *i* to *j*; on the contrary, the smaller the absolute value of *S*_*ij*_, the lower the degree of sensitivity.

### Study case and data sources

As the most populous developing country globally, China’s food security issues have attracted worldwide attention [44–49]. Since the reform and opening up 40 years ago, China’s total food production capacity has made great progress. The Chinese have used approximately 9% of the world’s arable land to feed 25% of the world’s population. However, in the long run, China’s food production faces several challenges: arable land resource reduction, severe water shortages, uncertain yield growth potential, and rising food production costs [45]. Growth in the population and diet transition promotes rigid growth in food demand [44, 46]. China’s overall food supply and demand are difficult to balance, and there will be a large gap in the supply of some agricultural products, particularly feed grains [44, 48]. China changed from being a net grain exporter to a net grain importer in 2010 [44] and has been the largest grain exporter country since 2013. The scale of grain imports increased to 108 million in 2018 [50]. Overreliance on the international market exposes China’s food security to many uncertain risks and increases China’s food security vulnerability. For example, from 2018 to 2020, the Sino-US trade friction has caused substantial fluctuations in the Sino-US grain trade, and the export restrictions imposed by supplier countries such as Russia, Vietnam, and Ukraine during the pandemic of the new crown epidemic in 2020 have interrupted global food supply chains and put tremendous pressure on China’s food security [13]. The above problems indicate that broadening China’s grain import channels and optimizing China’s overseas agricultural cooperation layout are urgent tasks facing the construction of a national food security strategy in the new era [45].

Based on the two theoretical hypotheses and mathematical models above, this study uses the grain trade matrix data of China and its partners to measure the vulnerability and sensitivity of China to each country, compare their differences, and visually display this spatial difference on the map using ArcGIS software. The grain trade matrix data between China and countries in 2014 and 2018 were collected from the General Administration of Customs of the People’s Republic of China(www.customs.gov.cn) [50]. The data used in this study are derived from the findings of internationally recognized and widely accepted scholars. In 2013, China’s rice, wheat, and maize were accessed at the net import stage. Therefore, the data after 2014 reflect only the characteristics of the era when China’s three kinds of staples synchronously net imports. The China customs database has recorded the grain import and export relationship matrix between China and 47 other countries or regions since 2014, including the European Union, China, Hong Kong, China Taiwan, and China Macao. Our study was conducted on a country-by-country basis, so we excluded the four regions to obtain 43 subjects.

## Results

### China’s import dependence on different exporters

In 2018, China imported US $5,734,027 worth of grain from 43 countries, including the United States, Australia, Vietnam, Thailand, Canada, Ukraine, France, Pakistan, Kazakhstan, and Argentina. The trade volume between China and each country is unbalanced. In economics, the market share of the first four trading entities is usually used to measure market concentration. If its market share is greater than 40%, it means that the market concentration is high, and there is a certain degree of market control [51]. Grain imports from the top four countries accounted for 67.32% of China’s total grain imports in 2018, which indicates a high concentration of China’s grain imports. However, compared to 2014, China’s grain import sources decreased by about 10 percent.

We focus on China’s degree of import dependence on each trading partner. We adopt the percentage of China’s total grain imports to measure the degree of China’s dependence on its grain export partners. These countries can be divided into four groups: highest export dependence, higher export dependence, moderate export dependence, and lower export dependence (Table 1).

**Table 1:** Grain trade interdependence between China and other countries.

Classification standard	Import dependence	Export dependence
Highest dependence (> = 15%)	Australia, the United States, Canada	Australia, Vietnam
Higher dependence (5%~15%)	Vietnam, Ukraine, Thailand	Canada, Ukraine, Thailand, Pakistan, Kazakhstan, Myanmar
Moderate dependence (1%~5%)	Pakistan, France, Kazakhstan, Myanmar	America, France, Japan
Lower dependence (< = 1%)	Russia, Germany, Japan, Argentina, Chile, Denmark, South Korea, India, the Netherlands, etc.	Russia, Chile, Germany, South Korea, Denmark, Philippines, Malaysia, Argentina, Netherlands, India

This analysis shows that the highest import dependence values are distributed in Australia, the USA, and Canada. According to recent data, Australia is China’s largest source of grain imports, and China’s import dependence on Australia is up to 23.52%. Although Sino-US trade friction results in an apparent decline in grain trade volume between them, it is currently difficult for China to find a substitute market for the United States, and its dependence is still as high as 15.85%. The grain export structure of Canada is similar to that of the United States, mainly corn and wheat. During the shrinking grain trade between China and the United States, the grain trade between China and Canada increased rapidly, and its dependence on it increased from 5.07% in 2014 to 15.05% in 2018.

The results show that the higher import dependence values were distributed in three emerging grain-exporting countries: Vietnam, Ukraine, and Thailand. Ukraine is the “second-largest granary” in Europe. It has continuously expanded grain trade with China since it signed agricultural cooperation agreements in 2012. In 2018, China’s grain imports from Ukraine reached US$ 731.964 million, and its dependence on imports increased to 12.77%. It is no exaggeration to say that Ukraine is most likely to replace the United States in the future, especially in the corn trade. Vietnam and Thailand are the first and second rice exporting countries in the world, and the two most important sources of rice import for China. China’s import dependence on them was 9.98% and 6.81%, respectively.

The results show that moderate import dependence values were distributed in Pakistan, France, Kazakhstan, and Myanmar. France was the largest source of grain imports for China in Europe, but China’s dependence on it has declined due to the competition for grain trade in the Black Sea region. Pakistan and Myanmar are adjacent to China, which is convenient for border trade. Pakistan’s staple food is wheat, while rice is mainly used for exports to earn foreign exchange. The price of Pakistan’s rice is low, and it is suitable for China to process raw materials such as monosodium glutamate and pastry. Myanmar’s high-quality rice has a high reputation in China, and China imports about 100000 tons of high-quality rice from Myanmar through border ports every year.

The results show that lower values of import dependence were distributed in the left couriers, including Russia, Germany, Japan, Argentina, Chile, Denmark, South Korea, India, and the Netherlands. China’s import dependence on these countries was less than 1%. It is worth mentioning that China’s grain imports from Russia were US $29.302 million in 2018, the highest in history, but it was only equivalent to 0.51% of China’s total grain imports. Even with soybeans, China’s dependence on Russia was no more than 4%. Compared with other commodity trades, China and Russia’s grain trade developed slowly, but there is much room for growth in the future.

### The partner’s export dependence on China

China has become the largest grain import market in the world. These countries export grain to China and form an export dependence on China. We adopt export dependence (*E*_*ij*_) to measure the degree of grain export dependence on China. These countries can be divided into four groups based on their value: highest export dependence, higher export dependence, moderate export dependence, and lower export dependence (Table 1).

The highest export dependence index groupings were found in Australia and Vietnam. With its grain output and per capita grain output high, Australia’s export pressure is high, and its grain shipped to China, Japan, and Europe. However, due to its closer distance to China, its grain exports are highly dependent on China, accounting for 27.93%. Similarly, China is Vietnam’s largest rice export market, and its dependence on China is more than 20%.

Higher export dependence index groupings are found in Canada, Ukraine, Thailand, Pakistan, Kazakhstan, and Myanmar. In 2014, Canada’s grain export to China was only $ 321.236 million, but it increased to 90451.6 dollars in 2018, and its export dependence on China also increased to 11.73%, an increase of 8.13% compared with 2014. As China advanced agricultural trade cooperation with countries along "the Belt and Road Initiative," the grain export trade of Ukraine, Thailand, Pakistan, Kazakhstan, and Myanmar to China continued to expand. Their export dependence in China was over 5%. Concerning Ukraine, China replaced Russia as the largest trading country in Ukraine. As in agri-trade, Ukraine mainly exports maize and wheat to China, and its dependence on China has reached 10.01%.

Moderate export dependence index groupings are found in the United States, France, and Japan. The U.S. grain export market is vast, and its grain exports to China account for only 4.29% of its total exports. Compared with China’s dependence on the United States, the U.S. dependence on China is relatively low, and it has been on a downward trend in recent years. France is the largest grain-producing and trading country in Europe. Its grain is mainly exported to EU countries and Central Asia, and its dependence on China is only 1.88%.

Lower export dependence index groupings are found in Russia, Chile, Germany, South Korea, Denmark, the Philippines, Malaysia, Argentina, the Netherlands, and India. The country’s export dependence on China was below 1%. The grain export scale of the above countries is small, and the volume of trade between China is small, except for Russia. Russia is currently the world’s largest wheat exporter, but its grain export markets are mainly in African and Southeast Asian countries. In 2018, Russia’s total grain exports reached US $10530.07 million, of which only 0.28% was exported to China. Therefore, Russia’s export dependence on China is lower.

### Spatial-temporal pattern evolution of the vulnerability of China’s grain trade

To analyze and characterize the spatial differences in China’s grain trade vulnerability, we applied the natural fracture method in ArcGIS software to divide the vulnerability index *V*_*ij*_ into three county-level categories across the world. These categories are referred to as higher, medium, and lower, respectively (Table 2).

**Table 2:** Classification for *V_ij_*.

Vulnerability level	Higher	Medium	Lower
*V_ij_* value range	(1.6, + ∞)	[0.7,1.6]	(- ∞,0.7)

**Fig 1:**
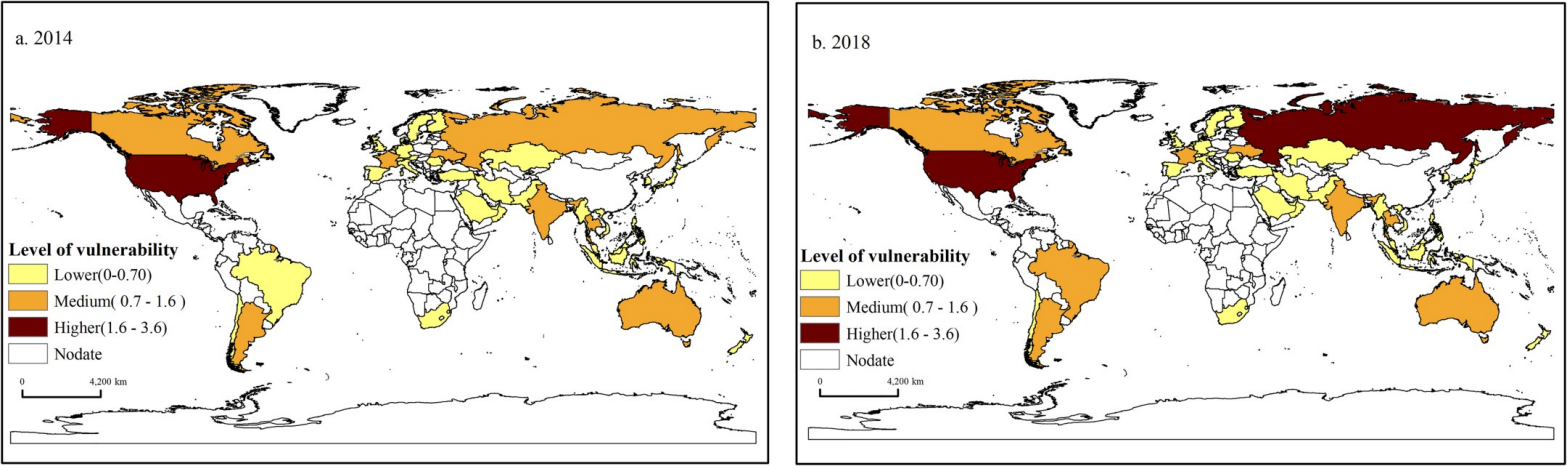
Spatial pattern of the vulnerability of China’s grain trade in 2014 and 2018.

Fig 1 indicates that in 2014, areas with higher vulnerability were concentrated in North America, mainly the United States. Medium vulnerable areas include eight countries, accounting for 18.60% of the total sources of China’s grain imports. It forms a spatial pattern of contiguous distribution, highly concentrated in Northeast Asia, North America, Southeast Asia, Oceania, South America, and sporadic distribution in Europe.

The lower vulnerable areas include 33 countries, accounting for 76% of the total sources of China’s grain imports, which are mainly distributed in Europe, Central Asia, Southeast Asia, and South America, and sporadically distributed in Africa and Oceania.

Compared with 2014, the scope of China’s grain trade’s higher and medium-vulnerability expanded in 2018, while the scope of lower vulnerability narrowed down. During 2014 and 2018, China’s higher vulnerability zone expanded rapidly, spreading from North America to Northeast Asia, and Russia changed from a medium-vulnerability zone to a higher vulnerability zone. The expansion of the medium-vulnerability zone is mainly due to Brazil’s transformation from a lower vulnerability zone to a medium-vulnerability zone. The narrowed down region of lower vulnerability occurred mainly in South America, while the other lower vulnerability area coverage remained. This change indicates that the external situation of China’s grain trade is more severe.

### Spatial-temporal pattern of grain trade sensitivity of China

To analyze and characterize the spatial differences in China’s grain trade sensitivity, we draw on Du’s research [35] by dividing the sensitivity indexes into five categories. The absolute value of the S_ij_ between China and other countries can be divided into three categories: higher sensitivity, medium sensitivity, and lower sensitivity. Higher sensitivity was then divided into high positive sensitivity and high negative sensitivity. The medium sensitivity was divided into positive and negative medium sensitivity according to their value’s positive and negative attributes. The specific classifications of sensitivity are presented in Table 3.

**Table 3:** Classification for *S_ij_*.

Sensitivity level	Negative higher sensitive	Medium negative sensitive	Lower sensitive	Positive medium sensitive	Positive higher sensitive
*S_ij_* value range	(-∞, -5]	(-5, -1]	(-1,1)	[1,5)	[5, + ∞)

**Fig 2:**
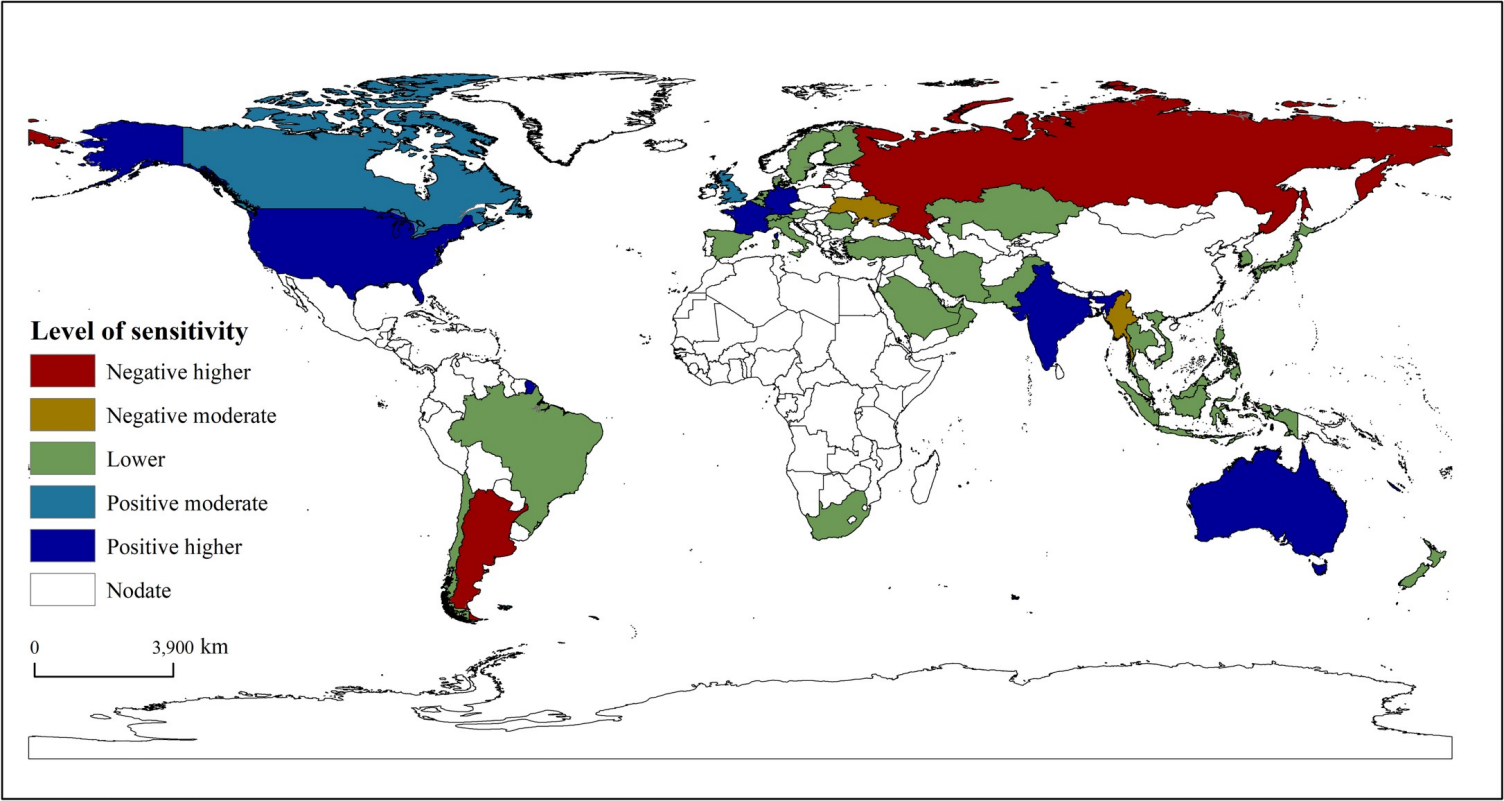
Sensitivity of China to its grain trade patterns.

Fig 2 and Table 4 show the higher sensitivity values distributed in Russia, Argentina, the United States, France, Germany, India, and Australia. China had a negative hypersensitivity to Russia in the bilateral grain trade. Compared to 2014, China’s total grain imports decreased in 2018, while Russia’s total grain export trade and bilateral grain trade between China and Russia increased by 47.51% and 254.06%, respectively. Similarly, China also had a negative hypersensitivity to Argentina, and the bilateral grain trade between China and Argentina decreased by 98.48%. On the contrary, China showed positive hypersensitivity to the United States, France, Germany, India, and Australia.

**Table 4:** Differences of China’s sensitivity to importing countries.

Sensitivity level	*S_ci_* value range	Countries
Negative higher sensitivity	< = -5	Russia (- 10.57), Argentina (- 6.88)
Negative moderate sensitivity	(-5~-1)	Myanmar (- 3.08), Ukraine (- 2.16)
Lower sensitivity	(-1~1)	Brazil (- 0.58), Vietnam (- 0.36), Kazakhstan (- 0.18), Pakistan (- 0.12), Japan (- 0.06), Thailand (- 0.05), Malaysia (0.01), Philippines (0.02), New Zealand (0.09), Italy (0.35), Austria (0.38), Finland (0.38), Denmark (0.53), Chile (0.55), etc.
Positive moderate sensitivity	(1~5)	Britain (2.62) and Canada (4.87)
Positive higher sensitivity	> = 5	America (5.67), France (5.73), Germany (5.85), India (7.38), and Australia (8.17).

The results show moderate values of sensitivity distributed in Britain, Canada, Myanmar, and Ukraine. China is moderately sensitive to Myanmar and Ukraine in the grain trade. Compared to 2014, China’s total grain imports decreased in 2018, but the volume of bilateral grain trade between China increased by 151.82% and 301.66%, respectively. Canada’s total grain export volume decreased by 17.53%, and the bilateral grain trade between China and Canada increased by 168.56%, indicating that China was moderately sensitive to Canada. The volume of bilateral grain trade between Britain and China is very small, but it has declined significantly (Fig 2 and Table 4).

The results show lower sensitivity values in Brazil, Vietnam, Kazakhstan, Pakistan, Japan, Thailand, Malaysia, Philippines, New Zealand, Italy, Austria, Finland, Denmark, and Chile. The sensitivity index values were between −1 and 1. Except for Vietnam, Kazakhstan, Pakistan, and Vietnam, the volume of bilateral trade in most countries was small (Fig 2 and Table 4).

## Conclusions

This article provides a novel analysis of the spatial-temporal pattern evolution of vulnerability and sensitivity of China to its 43 trade partners during the 2014–2018 period. Our results indicate that the degree of interdependence between China and its grain trading partners is asymmetric, generating trade vulnerability or economic power. For most trading partners, their export dependence on China is higher than China’s import dependence on them; for large grain-exporting countries such as Australia, the United States, and Canada, China’s import dependence on them is higher than their dependence on China.

Our findings indicate that the vulnerability of China’s food trade shows high spatiotemporal heterogeneity among countries. Medium vulnerable areas include eight countries and their distribution in Northeast Asia, North America, Southeast Asia, Oceania, South America, and Europe. The lower vulnerability area has the widest range and is widely distributed in Europe, Central Asia, Southeast Asia, South America, Africa, and Oceania. Compared with 2014, the scope of China’s grain trade’s higher and medium-vulnerability expanded in 2018, while the scope of lower vulnerability narrowed down. The higher vulnerability zone of China’s grain trade was smaller but expanded rapidly.

Our results also reveal that China has different sensitivities to fluctuations in grain markets in different countries. Specifically, China is highly sensitive to trade fluctuations in Russia, Argentina, the United States, France, Germany, India, and Australia, moderately sensitive to the United Kingdom, Canada, Myanmar, and Ukraine, and has low sensitivity to Brazil, Vietnam, Kazakhstan, Pakistan, Japan, Thailand, Malaysia, the Philippines, New Zealand, Italy, Austria, Finland, Denmark, and Chile.

The main contribution of this paper is the development of a methodology for food trade vulnerability assessment and examines the influence of international food trade on food security in importing countries, measured using the vulnerability index and sensitivity index. Our study also enables further analyses of these aspects to broaden and deepen our understanding of how globalized trade has influenced food security in developing countries. The results also have important implications for policymakers. This study explores China’s vulnerability and sensitivity to its grain trade partners, which can help contribute to a deeper understanding of the grain trade cooperation between China and other countries and provide theoretical and practical guidance for China to optimize grain import patterns. Indeed, policymakers could use this model to identify specific trade partners of the target to seek further cooperation. Nevertheless, this study only covers a relatively short period, from 2014 to 2018, owing to data limitations. Future studies should involve long time-series research in the case of data availability.

We greatly appreciate the helpful comments of the reviewers and editors, which have significantly contributed to improving the quality of the paper.

## Supporting information

S1 Fig Spatial pattern of the vulnerability of China’s grain trade in 2014 and 2018. (TIF)

S2 Fig Sensitivity of China to its grain trade patterns. (TIF)

S1 Table Grain trade interdependence between China and other countries. (PDF)

S2 Table Classification for *V_ij_*. (PDF)

S3 Table Classification for *S_ij_*. (PDF)

S4 Table Differences of China’s sensitivity to importing countries. (PDF)

S1 File Data. (ZIP)
