# Correction: Trade vulnerability assessment in the grain-importing countries: A case study of China

**DOI:** 10.1371/journal.pone.0316587

**Published:** 2024-12-26

**Authors:** The PLOS ONE Staff

The second author’s name is spelled incorrectly. The correct name is: Yong Xu. The correct citation is: Duan J, Xu Y, Jiang H (2021) Trade vulnerability assessment in the grain-importing countries: A case study of China. PLOS ONE 16(10): e0257987. https://doi.org/10.1371/journal.pone.0257987.

In the Conclusion section, the fifth paragraph should not have been indicated.

The publisher apologizes for the errors.
