# Supplementary figures and images for: Trade vulnerability assessment in the grain-importing countries: A case study of China
Source: PLoS One. 2021 Oct 22;16(10):e0257987. doi: 10.1371/journal.pone.0257987 (PMC8535458; doi:10.1371/journal.pone.0257987)

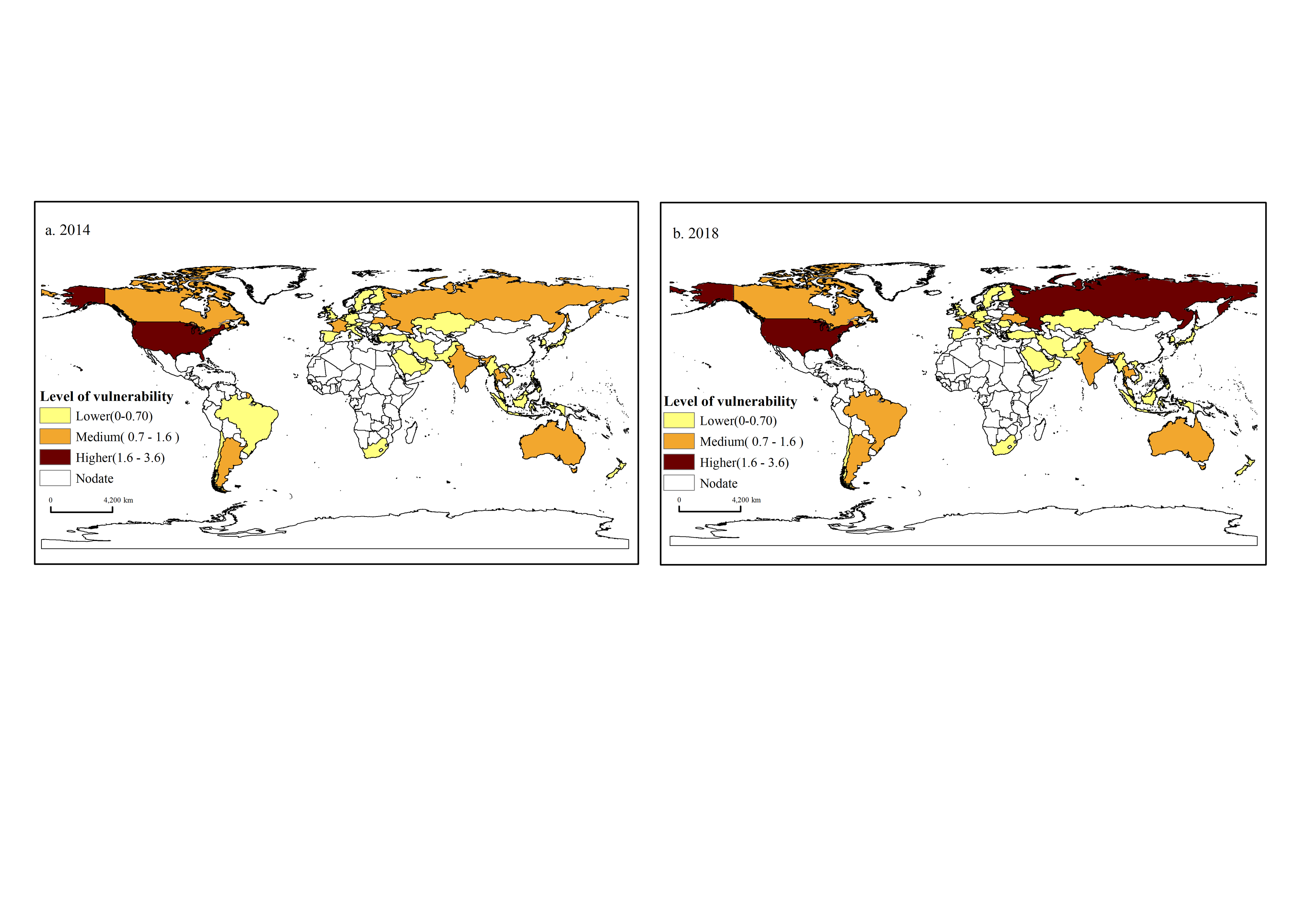

Supplement: S1 Fig — (TIF) [file pone.0257987.s001.tif]

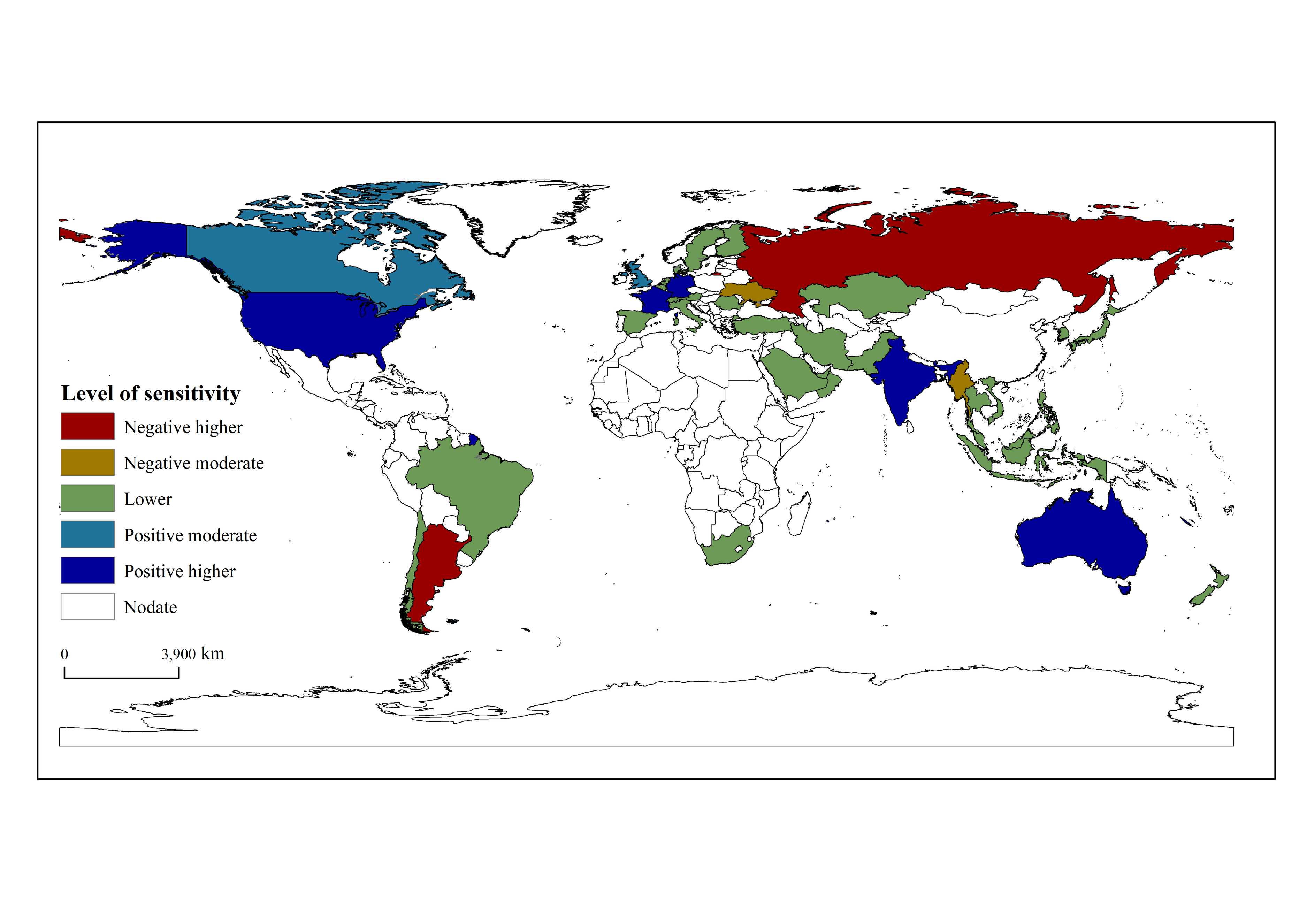

Supplement: S2 Fig — (TIF) [file pone.0257987.s002.tif]
