# Supplementary material for: Trade vulnerability assessment in the grain-importing countries: A case study of China
Source: PLoS One. 2021 Oct 22;16(10):e0257987. doi: 10.1371/journal.pone.0257987 (PMC8535458; doi:10.1371/journal.pone.0257987)
Supplement: S1 Table — (PDF) [file pone.0257987.s003.pdf]

Table1. Grain trade interdependence between China and other countries

Classification standard	Import dependence	Export dependence
Highest dependence (>=15%)	Australia, the United States, Canada	Australia, Vietnam
Higher dependence (5%~15%)	Vietnam, Ukraine, Thailand	Canada, Ukraine, Thailand, Pakistan, Kazakhstan, Myanmar
Moderate dependence (1%~5%)	Pakistan, France, Kazakhstan, Myanmar	America, France, Japan
Lower dependence (<=1%)	Russia, Germany, Japan, Argentina, Chile, Denmark, South Korea, India, the Netherlands, etc.	Russia, Chile, Germany, South Korea, Denmark, Philippines, Malaysia, Argentina, Netherlands, India
