# Supplementary material for: Trade vulnerability assessment in the grain-importing countries: A case study of China
Source: PLoS One. 2021 Oct 22;16(10):e0257987. doi: 10.1371/journal.pone.0257987 (PMC8535458; doi:10.1371/journal.pone.0257987)
Supplement: S2 Table — (PDF) [file pone.0257987.s004.pdf]

Table2. Classification for V_{ij}

Vulnerability level	Higher	Medium	Lower
V_{ij} value range	(1.6, + ∞)	[0.7,1.6]	(- ∞,0.7)
