# Supplementary material for: Trade vulnerability assessment in the grain-importing countries: A case study of China
Source: PLoS One. 2021 Oct 22;16(10):e0257987. doi: 10.1371/journal.pone.0257987 (PMC8535458; doi:10.1371/journal.pone.0257987)
Supplement: S3 Table — (PDF) [file pone.0257987.s005.pdf]

**Table3. Classification for $S_{ij}$**

| Sensitivity level | Negative higher sensitive | Medium negative sensitive | Lower sensitive | Positive medium sensitive | Positive higher sensitive |
|---|---|---|---|---|---|
| $S_{ij}$ value range | (-∞, -5] | (-5, -1] | (-1,1) | [1,5) | [5, + ∞) |
