# Supplementary material for: Trade vulnerability assessment in the grain-importing countries: A case study of China
Source: PLoS One. 2021 Oct 22;16(10):e0257987. doi: 10.1371/journal.pone.0257987 (PMC8535458; doi:10.1371/journal.pone.0257987)
Supplement: S4 Table — (PDF) [file pone.0257987.s006.pdf]

Table4. Differences of China's sensitivity to importing countries

Sensitivity level	S_{ci} value range	Countries
Negative higher sensitivity	<=-5	Russia (- 10.57), Argentina (- 6.88)
Negative moderate sensitivity	(-5~-1)	Myanmar (- 3.08), Ukraine (- 2.16)
Lower sensitivity	(-1~1)	Brazil (- 0.58), Vietnam (- 0.36), Kazakhstan (- 0.18), Pakistan (- 0.12), Japan (- 0.06), Thailand (- 0.05), Malaysia (0.01), Philippines (0.02), New Zealand (0.09), Italy (0.35), Austria (0.38), Finland (0.38), Denmark (0.53), Chile (0.55), etc.
Positive moderate sensitivity	(1~5)	Britain (2.62) and Canada (4.87)
Positive higher sensitivity	>= 5	America (5.67), France (5.73), Germany (5.85), India (7.38), and Australia (8.17).
